# Eosinophils from Murine Lamina Propria Induce Differentiation of Naïve T Cells into Regulatory T Cells via TGF-β1 and Retinoic Acid

**DOI:** 10.1371/journal.pone.0142881

**Published:** 2015-11-20

**Authors:** Hong-Hu Chen, Ai-Hua Sun, David M. Ojcius, Wei-Lin Hu, Yu-Mei Ge, Xu’ai Lin, Lan-Juan Li, Jian-Ping Pan, Jie Yan

**Affiliations:** 1 Collaborative Innovation Center for Diagnosis and Treatment of Infectious Diseases, The First Affiliated Hospital, Zhejiang University School of Medicine, Hangzhou, Zhejiang, 310003, P.R. China; 2 Division of Basic Medical Microbiology, State Key Laboratory for Diagnosis and Treatment of Infectious Diseases, The First Affiliated Hospital, Zhejiang University School of Medicine, Hangzhou, Zhejiang, 310058, P.R. China; 3 Department of Medical Microbiology and Parasitology, Zhejiang University School of Medicine, Hangzhou, Zhejiang, 310058, P.R. China; 4 Zhejiang Provincial Center for Disease Control and Prevention, Hangzhou, Zhejiang, 310051, P.R.China; 5 Faculty of Basic Medicine, Zhejiang Medical College, Hangzhou, Zhejiang, 310053, P.R. China; 6 Health Sciences Research Institute and Molecular Cell Biology, University of California, Merced, California, 95343, United States of America; 7 Department of Clinical Medicine, School of Medicine, Zhejiang University City College, Hangzhou, Zhejiang, 310015, P.R. China; Mie University Graduate School of Medicine, JAPAN

## Abstract

Treg cells play a crucial role in immune tolerance, but mechanisms that induce Treg cells are poorly understood. We here have described eosinophils in lamina propria (LP) that displayed high aldehyde dehydrogenase (ALDH) activity, a rate-limiting step during all-trans retinoic acid (ATRA) synthesis, and expressed TGF-β1 mRNA and high levels of ATRA. Co-incubation assay confirmed that LP eosinophils induced the differentiation of naïve T cells into Treg cells. Differentiation promoted by LP eosinophils were inhibited by blocked either TGF-β1 or ATRA. Peripheral blood (PB) eosinophils did not produce ATRA and could not induce Treg differentiation. These data identifies LP eosinophils as effective inducers of Treg cell differentiation through a mechanism dependent on TGF-β1 and ATRA.

## Introduction

The immune system needs to carefully balance both immune responses and immune tolerance[1]. Immune responses eliminate harmful antigens such as pathogens and dead or aberrant host cells[1, 2]. At the same time, immune tolerance is needed in order to avoid damaging normal host tissues and to allow the presence of harmless antigens such as commensal bacteria and food antigens in the intestinal tract[3].

Regulatory T (Treg) cells play a crucial role in generation and maintenance of immune tolerance[4]. It has been shown that transforming growth factor-beta (TGF-β) stimulates naïve CD4^+^CD25^−^ T cells to differentiate into either CD4^+^CD25^+^Foxp3^+^ Treg cells or Th17 cells[5, 6], while all-trans retinoic acid (ATRA) from intestinal dendritic cells (DC) induces the differentiation of naïve T cells into Treg cells in presence of TGF-β1 but suppress the differentiation of Th1, Th2 and Th17 cells[7–9]. The activation of aldehyde dehydrogenase (ALDH), a unique rate-limiting enzyme during ATRA synthesis, has been considered as the signal for cells to produce ATRA[10].

The mucosal immune system rather than systemic immune system acts as the main sensor and effector in responses to exogenous antigens[11]. Gut-associated lymphoid tissue (GALT), the largest lymphoid organ in the mucosal immune system, is comprised of Peyer’s patches, interdigitating lymphocytes, plasma cells and lymphocytes in the LP, and mesenteric lymph nodes, in which LP is the loci for the highest frequency of Treg cells expansion[12]. In LP, CD103^+^ DC and CD11b^+^ F4/80^+^ CD11c^−^ macrophages can induce the generation of Treg cells, while CD11c^+^ CD11b^+^ CD103^−^ DC induce the differentiation of Th17 cells[13, 14]. However, except for DC and macrophages, no other immune cells have been reported to induce the differentiation of naïve CD4^+^CD25^−^ T cells.

While investigating the function of DC and macrophages from murine LP, we identified eosinophils that displayed a high activity of ALDH as well as producing high levels of ATRA and expressing TGF-β1 mRNA. In the present study, we provided evidence that this subset of eosinophils (LP eosinophils) represents a novel inducer of the differentiation of naïve T cells into Treg cells.

## Materials and Methods

### Mice

Female wild-type or OT-II transgenic C57BL/6 mice of 6–10 weeks of age were kindly provided by Joint Venture SIPPR-BK Experimental Animal Company (Shanghai, China) or Dr. Jian-Li Wang (Department of Immunology, Zhejiang University School of Medicine, China).

All mice were maintained in a specific pathogen-free animal facility with a standardized light (12 h light/dark cycle), temperature (22±1°C) and humidity (55±15%). Animals were fed food and water freely. Cages were changed weekly. At this study, mice were sacrificed by cervical dislocation. All of animal experimental protocols were approved by the Ethics Committee for Animal Experiment of Zhejiang University.

### Cells

To isolate LP cells, small intestines were removed and their Peyer's patches were cleaned, then opened along the mesenteric side and washed of fecal contents. Intestines were cut into 5 mm in length and incubated for 30 min at 37°C with PBS containing 10% FCS, 10 mM EDTA, 20 mM HEPES, 100 U/ml penicillin, and 100 μg/ml streptomycin (Gibco, USA) to remove the epithelium. Tissues were washed twice with PBS, minced, and digested for 60 min with continuous stirring at 37°C with 1 mg/ml collagenase D (Roche, Germany) and 0.1 mg/ml Dnase (Sigma, USA) in RPMI 1640 plus 10% FCS. Tissues were filtered through 40 μm and 70 μm cell strainer (BD Biosciences, USA) and washed in PBS twice. Cells were resuspended into FACS buffer and stained with biotin-conjugated monoclonal anti-mouse CD11c (N418;), anti-mouse CD11b (M1/70) (eBioscience, USA), rat anti-mouse Siglec-F (E50-2440; BD Pharmingen, USA), anti-mouse MHC-II (AF6-120.1), anti-mouse DEC-205 (205yekta), anti-mouse CD103 (2E7), anti-mouse CD40 (1C10), anti-mouse CD80 (16-10A1), anti-mouse CD86 (GL-1), and anti-mouse F4/80 (BM8)(eBioscience). The data were analyzed using CELLQuest (BD Biosciences) and FlowJo (TreeStar, USA) software. In the four sorted cell subsets (P1-P4), the P4 subset displayed an eosinophil-specific phenotype such as positive Siglec-F; this cell subset was therefore isolated from the single-cell suspension using CD11b-coated microbeads (Miltenyi Biotec, Germany) and the CD11b^+^cells were then sorted using a FACSAria II flow cytometer (BD Biosciences) with FITC-conjugated rat anti-mouse CCR3 (83101; R&D, USA) and PE-conjugated rat anti-mouse Siglec-F (E50-2440). In addition, the PB eosinophils of wild-type C57BL/6 mice were also isolated as above. CD11c^+^ MHC-II^+^ CD103^+^ LP DC isolated from the LP cells using CD11c-coated microbeads (Miltenyi Biotec) and flow cytometric sorting with PE-conjugated anti-mouse MHC-II (AF6-120.1) and APC-conjugated anti-mouse CD103 (2E7)(eBioscience). In functional experiments, anti-mouse CD16/CD32 (93; eBioscience) was used for blocking Fc receptors to avoid non-specific effects of the antibodies used.

### Detection of ALDH activity of LP cells

The aldehyde dehydrogenase (ALDH) activity of the LP cells was detected using an ALDEFLUOR staining kit (StemCell Technologies, Canada). Briefly, the LP cells in ALDEFLUOR buffer containing ALDH substrate were incubated at 37°C for 45 min in the dark. After washing with the buffer and centrifugation, the cell pellet was suspended in ice-cold ALDEFLUOR buffer, add and incubate with biotin-conjugated monoclonal anti-CD11c, anti-CD11b and anti-Siglec-F, and then detected by flow cytometry[15]. The ALDH activity was measured by the difference values of mean fluorescence intensity (MFI) of the LP cells before or after treatment with 300 μM ALDH inhibitor DEAB (StemCell Technologies).

### Morphological examination of LP eosinophils

May-Grunwald-Giemsa dye was used to stain LP eosinophils and observed under a light microscope as previously described[16].

### Determination of ATRA produced by LP and PB eosinophils

A LC(liquid chromatography)/MS/MS-based method was established for quantitative detection of ATRA in cell cultures referring to a previous report[17]. Briefly, 10^5^ CD11b^+^ Siglec-F^+^ CCR3^+^ LP or PB eosinophils or CD11c^+^MHC-II^+^CD103^+^ LP DC was mixed with 1 ml 0.025 M KOH-ethanol, and then added internal standard. The mixture was added with 10 ml hexane for vortex, followed by a short centrifugation for stratification. The lower aqueous phase was collected to mix with 60 μl of 4 M HCl and then added with hexane as above. After centrifugation, the hexane phase was collected to remove the solvent with a gentle nitrogen stream. The residue was dissolved in 100 μl acetonitrile for measurement of ATRA using a triple-quadrupole mass spectrometer (Agilent Technologies, USA). In the detection, acitretin (Sigma), an analogue of ATRA, was used as the internal reference[18].

### Real-time PCR

Total RNA was extracted from purified LP eosinophils, CD11c^+^MHC-II^+^CD103^+^ LP DC or PB eosinophils using RNeasy Mini Kit (Qiagen, Germany). cDNA was generated using the Omniscript RT kit (if starting material was >50 ng) or Sensiscript RT kit (if starting material was <50 ng) (Qiagen). cDNA was used as a template for quantitative real-time PCR using SYBR Green Master Mix (Applied Biosystems, USA), and the following gene-specific primers: *tgfb1* primers ACCATGCCAACTTCTGTCTG, CGGGTTGTGTTGGTTGTAGA; and *Gapdh* primers TGGCAAAGTGGAGATTGTTGCC, AAGATGGTGATGGGCTTCCCG. PCR was performed using an ABI 7500 Real-time PCR System (Applied Biosystems). Gene expression was calculated relative to *Gapdh*.

### Detection of LP and PB eosinophils-induced Treg response

Naïve T cells from splenocytes of OT-II transgenic C57BL/6 mice were isolated using a CD4^+^CD62L^+^ T cell isolation kit-II (Miltenyi Biotec). Eosinophils were cultured with 5 ng/mL recombinant murine GM-CSF (Peprotech, USA) to sustain their viability. The naïve T cells (10^5^ cells) were co-cultured with the LP eosinophils (10^5^ cells) isolated from wild-type C57BL/6 mice in 10% FCS RPMI-1640 medium containing 1.5 μg OVA peptide (ISQVHAAHAEINEAGR) and 10^4^ irradiated murine spleen CD11c^+^ cells at 37°C in an atmosphere of 5% CO_2_ for 4 d[14, 19]. In some experiments, the following were included in the coculture conditions: (a) Anti–human TGF-β1 (Peprotech) or rat IgG1 isotype control antibody (eBioscience) was added to cultures at a final concentration of 1 μg/ml. (b) Recombinant human TGF-β1 (Peprotech) at varying concentrations; and (c) RA receptor inhibitors LE540 (Wako Chemicals, Japan) and LE135 (Tocris Bioscience, UK), each at 1μM. The differentiated Treg cells in the 4 d co-cultures were identified by flow cytometry using FITC-conjugated anti-CD4 and APC-conjugated anti-Foxp3 in a Treg cell identification kit (eBioscience). At the same time, the ability of the PB eosinophils to induce a Treg response during co-cultivation was also determined as above.

### In vitro suppressor assay

To examine the suppressive capacity of LP eosinophil-induced Treg cells. LP eosinophil-induced Treg cells were generated in vitro and sorted to >99% purity. 10^5^ CFSE-labeled murine splenic CD4^+^ T cells were stimulated with 1 μl anti-CD3/CD28 coated beads (Invitrogen, USA), and the numbers of LP eosinophil-induced Treg cells indicated in the figures for 3 d.

## Results

### High ALDH expression on CD11c^int^ CD11b^hi^ LP eosinophils

**Fig 1:**
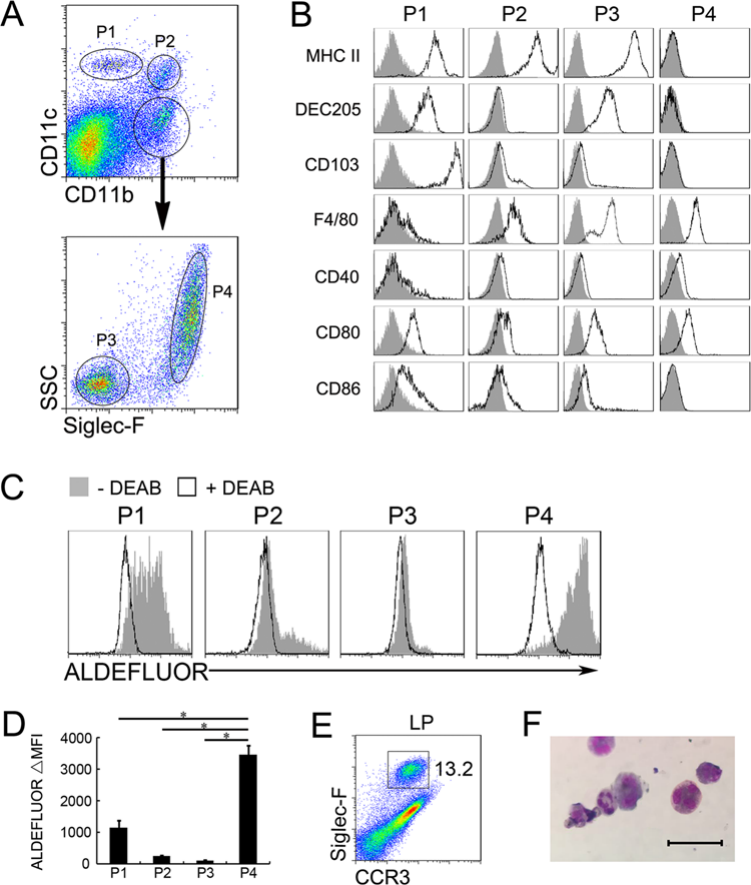
High ALDH expression on CD11c^int^ CD11b^hi^ LP eosinophils. (A) Cells from LP were stained with anti-mouse CD11c, CD11b and Siglec-F Abs, and then CD11c^+^ CD11b^−^ (P1), CD11c^+^ CD11b^+^ (P2), CD11c^−^ CD11b^+^ side scatter (SSC)^lo^ Siglec-F^−^ (P3) and CD11c^−^ CD11b^+^ SSC^hi^ Siglec-F^+^ cells (P4) were gated by flow cytometry. (B) Cells from LP were stained with a panel of Abs and the indicated surface molecules of four populations were analyzed by flow cytometry. Filled histograms are the isotype control.(C) Four subsets were sorted and ALDH activity of four subsets were determined by Aldefluor assay. (D) Statistical summary of ALDH activity of four subsets. Data from experiments such as that shown in C. Bars show the means ± SD of three independent experiments. (E) CD11b^+^ cells were isolated from the LP and stained with anti-mouse Siglec-F and CCR3 Abs, the percentage of Siglec-F^+^CCR3^+^ eosinophils were analyzed by flow cytometry.(F) May-Grunwald-Giemsa staining of sorted CD11b^+^Siglec-F^+^CCR3^+^ cells from LP. Scale bar, 20 μm.

Previous studies have identified CD11c^+^ CD11b^−^ DC (P1), CD11c^+^ CD11b^+^ DC (P2), CD11c^−^ CD11b^+^ side scatter (SSC)^lo^ Siglec-F^−^ macrophages (P3) and CD11c^−^ CD11b^+^ SSC^hi^ Siglec-F^+^ eosinophils (P4) in the cells from murine small intestinal LP (Fig 1A and 1B)[16, 20–22]. Of these four subsets from the LP of C57BL/6 mice, an Aldefluor assay confirmed that the CD11c^int^ CD11b^hi^ eosinophils had a significantly higher activity of ALDH, an indicator for the ability of the cells to produce ATRA[10], than the CD11c^hi^ CD11b^lo^ DC, CD11c^hi^ CD11b^hi^ DC and CD11c^int^ CD11b^int^ macrophages (Fig 1C and 1D). Large number of eosinophils present in normal LP[23, 24], and we found 13.2% of the total CD11b^+^ cells in the LP expressed CCR3 and Siglec-F, two markers of eosinophils [25, 26] (Fig 1E). Moreover, the CD11b^+^ Siglec-F^+^CCR3^+^ cells from LP presented a unique polymorphic nucleus and eosinophilic granules (Fig 1F), two common features of eosinophils. Thus, LP eosinophils maybe take an important role in mucosal immunity at steady state by secreting a higher level of ATRA.

### Quantitative measurement of ATRA by LC/MS/MS

**Fig 2:**
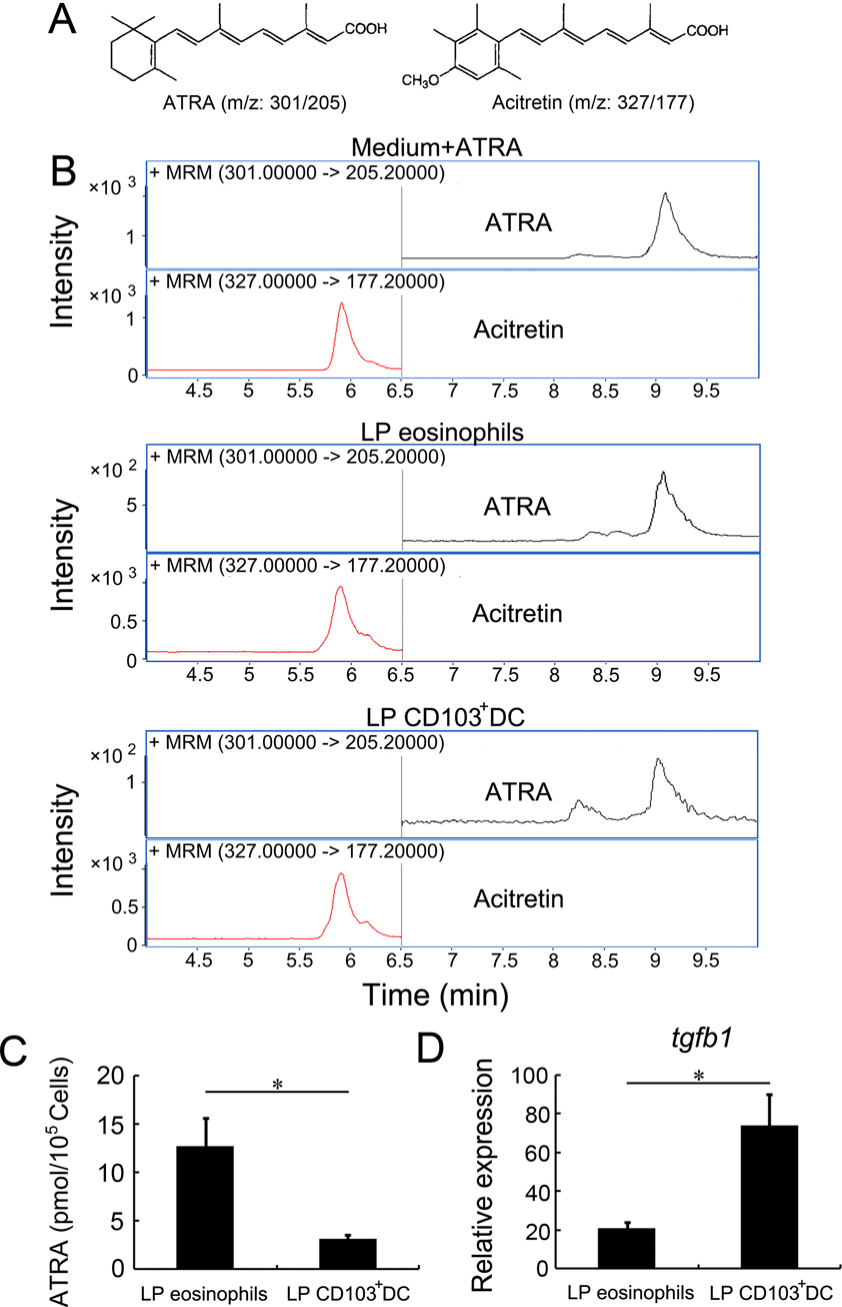
LP eosinophils produce ATRA and TGF-β1. (A) Structures of ATRA and acitretin. (B) LP eosinophils or CD11c^+^MHC-II^+^CD103^+^ LP DC were sorted and extracted. ATRA in LP eosinophils or CD11c^+^MHC-II^+^CD103^+^ LP DC were quantified by LC/MS/MS. The acitretin was used as the internal standard for ATRA quantification. (C) ATRA produced by LP eosinophils and CD11c^+^MHC-II^+^CD103^+^ LP DC. Bars show the means ± SD of three independent experiments. (D) RNA was isolated from purified LP eosinophils or CD11c^+^MHC-II^+^CD103^+^ LP DC, and expression of *tgfb1* were analyzed by quantitative real-time PCR. Values are expressed relative to *Gapdh*. Bars show the means ± SD of three independent experiments.

ATRA is a crucial factor during differentiation of naïve T cells into Treg cells[7]. In previous studies, CD11c^+^ MHC-II^+^ CD103^+^ LP DC was found to promote de novo generation of Foxp3 Treg cells via ATRA[13]. Therefore, to quantify ATRA produced by the CD11b^+^ Siglec-F^+^ CCR3^+^ LP eosinophils (the CD11c^−^ CD11b^+^ SSC^hi^ Siglec-F^+^ subset from the P4 in Fig 1A) or CD11c^+^MHC-II^+^CD103^+^ LP DC (P1), a LC/MS/MS-based method was established for quantitative detection of ATRA, in which acitretin, an analogue of ATRA, was used as the internal standard (Fig 2A)[17, 18]. The results showed that the LC/MS/MS method could accurately quantify the ATRA in LP eosinophils and CD11c^+^ MHC-II^+^ CD103^+^ LP DC (Fig 2B).

### ATRA and TGF-β1 produced by LP eosinophils

The LC/MS/MS demonstrated that the 10^5^ CD11b^+^ Siglec-F^+^ CCR3^+^ LP eosinophils could produce a higher level of ATRA (12.7±2.9 pmol/10^5^ cells), as the same number of CD11c^+^MHC-II^+^ CD103^+^ LP DC, which produced 3.1±0.5 pmol/10^5^ cells ATRA (Fig 2B and 2C). Besides ATRA[7], TGF-β1 has been shown to induce differentiation of naïve CD4^+^CD25^−^ T cells[5, 6, 27]. Analysis of gene expression by LP eosinophils revealed that LP eosinophils expressed *tgfb1* related to TGF-β1 (Fig 2D). The data suggested that the LP eosinophils have the potential to induce the differentiation of naïve T cells into Treg cells.

### Treg differentiation induced by LP eosinophils

**Fig 3:**
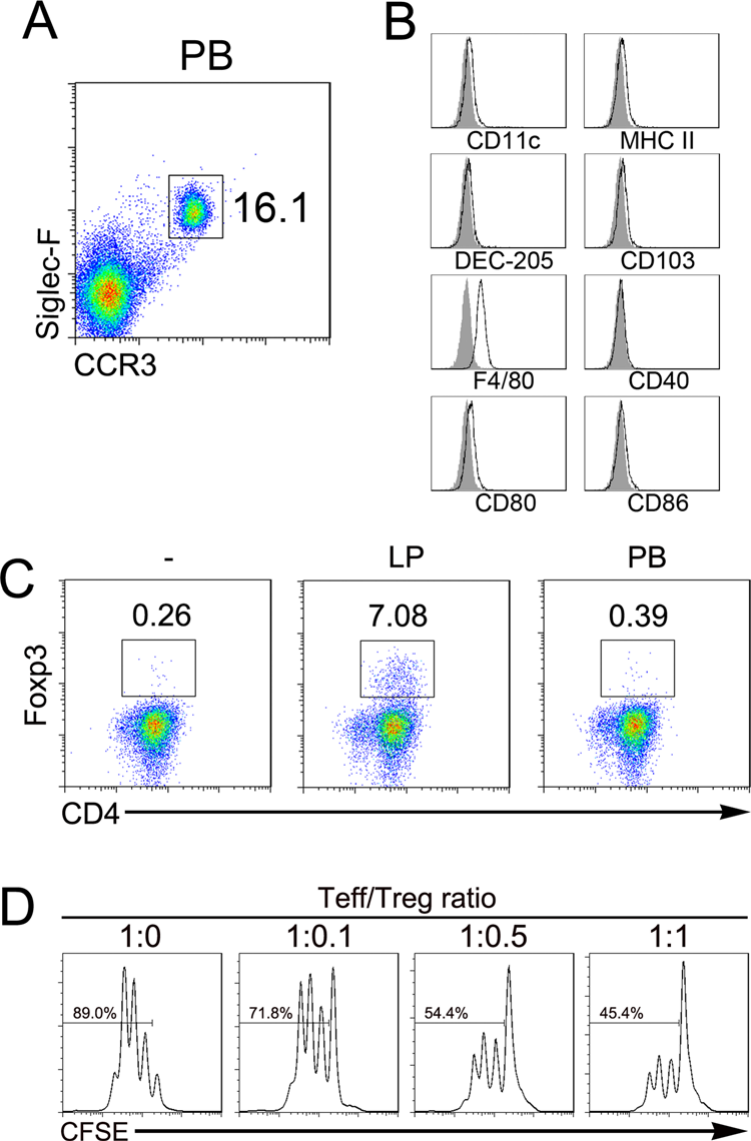
Induction of Foxp3^+^ Treg cells by LP eosinophils but not by PB eosinophils. (A) CD11b^+^ cells were isolated from the PB and stained with anti-mouse Siglec-F and CCR3 Abs, the percentage of Siglec-F^+^CCR3^+^ eosinophils were analyzed by flow cytometry. (B) Cells from BP were stained with a panel of Abs and the indicated surface molecules of the cells were analyzed by flow cytometry. Filled histograms are the isotype control. (C) MACS-sorted CD4^+^CD62L^+^ T cell from the OT-II mouse were cultured under activating coditions with LP or PB eosinophils, and Foxp3 expression by CD4^+^ T cells was analyzed by flow cytometry after 4 d of culture. (D) CFSE-labeled CD4^+^ T cells were cultured with sorted LP eosinophil-induced Treg at the indicated ratios (Teff:Treg×10^5^) under activating conditions for 3 d. The proliferation of CD4^+^ T cells was analyzed by flow cytometry.

We next examined whether Lp eosinophils could induce Treg cells conversion. To this end, eosinophils from the LP or PB were isolated (Figs 1E and 3A). Compared to the LP eosinophils (Fig 1B), the PB eosinophils also expressed F4/80 but did not express CD11c and CD80 (Fig 3B). However, neither the LP eosinophils nor the PB eosinophils expressed MHC II, DEC-205, CD86, CD40 and CD103. Purified OT-II CD4+ T cells, which express a transgenic T cell receptor specific for ovalbumin (OVA) peptide presented by major histocompatibility complex class II molecules, cultured together with LP or PB eosinophils in the presence of OVA peptide and irradiated DC [28]. After 4 d of co-incubation, we compared the ability of LP and PB eosinophils to induce expression of Foxp3 in CD4^+^ T cells, it was found that only LP eosinophils was able to induce expression of Foxp3 (Fig 3C). Next, we tested the suppressive capacity of LP eosinophil-induced Treg cells. The data show that LP eosinophil-induced Treg cells could suppress proliferation of T effector cells in vitro (Fig 3D).

### TGF-β1 enhances the conversion of Treg cells in the presence of Lp eosinophils

**Fig 4:**
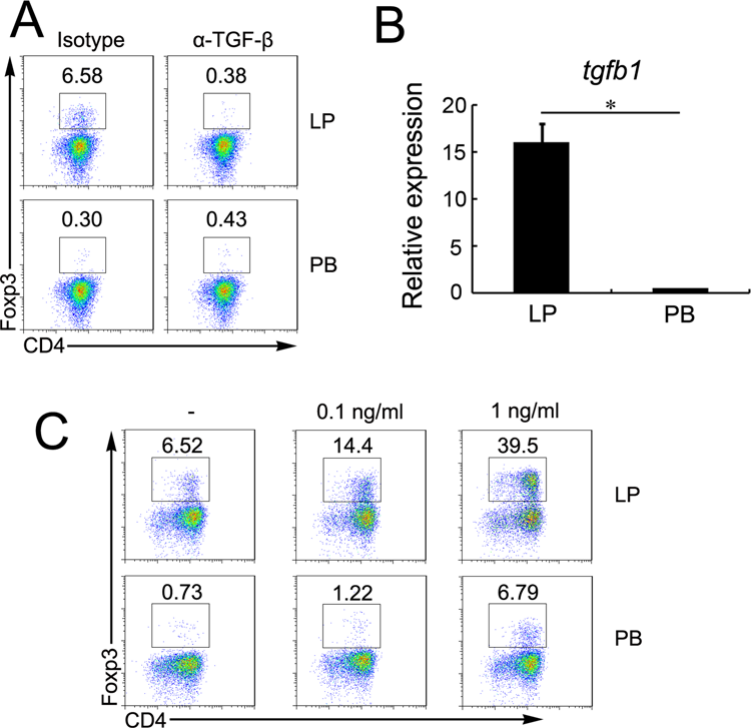
TGF-β1 enhances the conversion of Treg cells in the presence of Lp eosinophils. (A) MACS-sorted CD4^+^CD62L^+^ T cell from the OT-II mouse were cultured under activating coditions with LP or PB eosinophils as described in Fig 3 with IgG1 isotype control antibody (isotype) or TGF-β neutralizing antibody (α-TGF-β). The cells were stain for Foxp3 and CD4 and analyzed by flow cytometry. (B) RNA was isolated from purified LP or PB eosinophils, and expression of *tgfb1* were analyzed by quantitative real-time PCR. Values are expressed relative to *Gapdh*. Bars show the means ± SD of three independent experiments. (C) MACS-sorted CD4^+^CD62L^+^ T cell from the OT-II mouse were cultured under activating coditions with LP or PB eosinophils as described in Fig 3 with the indicated concentrations of TGF-β1. The cells were stain for Foxp3 and CD4 and analyzed by flow cytometry.

**Fig 5:**
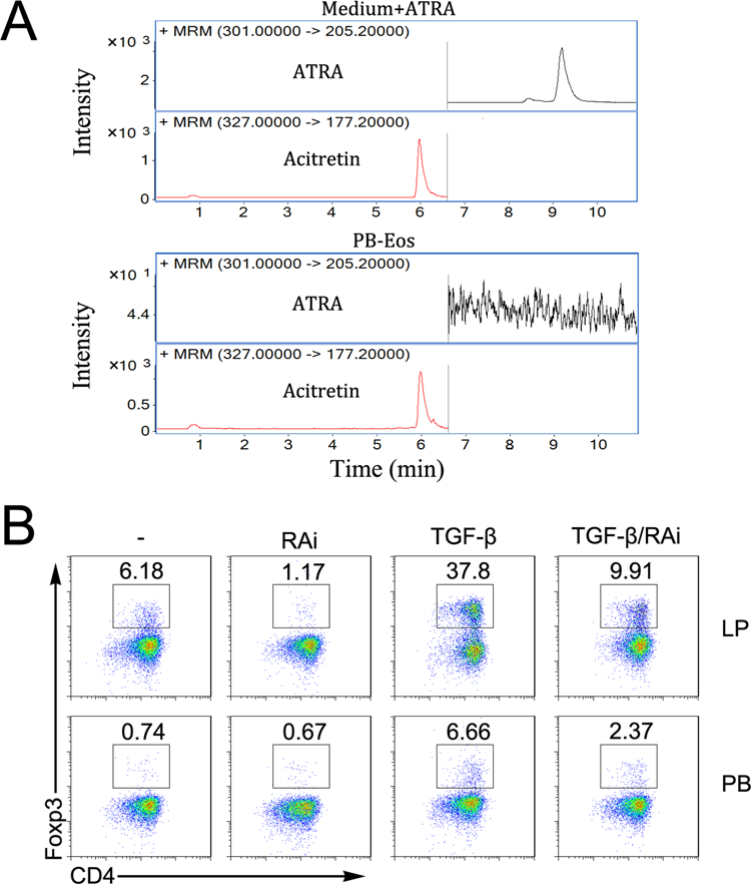
ATRA produced by Lp eosinophils is responsible for conversion of Treg cells. (A) BP eosinophils were sorted and extracted. ATRA in BP eosinophils were quantified by LC/MS/MS. The acitretin was used as the internal standard for ATRA quantification. (B) MACS-sorted CD4^+^CD62L^+^ T cell from the OT-II mouse were cultured under activating coditions with LP or PB eosinophils as described in Fig 3 in the absence (–) or presence of 1ng/ml TGF-β1, or the RA receptor antagonist LE135 and LE540 (RAi). CD4^+^Foxp3^+^ Treg cells differentiation was analyzed by intracellular staining and flow cytometry.

As we have shown that LP eosinophils express TGF-β1 mRNA, we analyzed whether TGF-β1 played any role in the development of Treg cells. Thus, naive T cells were again co-cultured with LP or PB eosinophils, with or without antibody neutralization of TGF-β1. Neutralization of TGF-β1 lead to a significant decrease in the frequency of Treg cells (6.58% vs 0.38%) (Fig 4A). Next, we asked whether the functional difference between LP and PB eosinophils was related to the ability of producing TGF-β1. LP eosinophils expressed higher levels of *tgfb1* (Fig 4B). To test whether TGF-β1 was able to exert an enhancing effect on Treg cells conversion, we added increasing doses of recombinant TGF-β1 to cultures. At low TGF-β1 concentrations (0.1 ng/ml), Treg cells conversion increased by 120% in LP eosinophils co-cultures (from 6.52 to 14.4%), and inclusion of 1 ng/ml TGF-β1 resulted in the expression of Foxp3 by ~40% of T cells (Fig 5C). Although PB eosinophils induce minor Foxp3 expression in the absence of exogenous TGF-β1, they remained much lower than LP eosinophils at inducing T reg cells (Fig 4C). The data suggested that the LP eosinophils have the ability to induce differentiation of naïve T cells into Treg cells *via* TGF-β1-dependent mechanisms.

### ATRA produced by Lp eosinophils is responsible for conversion of Treg cells

Differentiation of Treg cells is enhanced by the ATRA[7]. We have previously observed that the LP eosinophils could produce a high level of ATRA. And we now show that PB eosinophils cannot produce ATRA (Fig 5A). As a result, we investigated the function of the ATRA in this process. To assess whether ATRA was involved in the differentiation of TGF-β1-dependent Foxp3^+^ Treg cells induced by LP eosinophils, we added the synthetic ATRA receptor antagonist LE540 and LE135 (RAi) to co-cultures. The addition of RAi reduced the frequency of Foxp3^+^ Treg cells generated by LP eosinophils from 6.18% to 1.17%, and neutralization of ATRA inhibited Treg cells conversion by 74% and 64% in LP and PB eosinophils co-cultures in presence of TGF-β1 (Fig 5B). Therefore, the mechanisms by which LP eosinophils induced the generation of Treg cells involved ATRA.

## Discussion

Immune tolerance in the intestinal tract is required for the presence of the normal gut microbiota and the uptake of macromolecular nutrients from food[1, 3]. Although the mechanisms of immune tolerance are complicated, Treg cells are considered as the crucial inducers of immune tolerance[4, 5, 16]. It has been reported that CD4^+^CD25^+^Foxp3^+^ Treg cells could be induced by the CD103^+^ DC from mouse mesenteric lymph nodes (MLN) or intestinal LP *via* a TGF-β1- and ATRA-dependent mechanism[13, 19]. Denning *et al* confirmed that the CD11b^+^F4/80^+^CD11C¯ macrophages from the murine intestinal LP also have the ability to induce Treg cells during co-incubation of the macrophages with naïve T cells[14]. However, except for DC and macrophages, no other immune cells have been shown to possess the ability to induce Treg differentiation.

Uematsu *et al*. described an eosinophils subset expressing the F4/80 macrophage marker in murine colon LP (LP eosinophils)[16], but the function of LP eosinophils had not been characterized yet. In the present study, we observed LP eosinophils of C57BL/6 mice consistent with the eosinophils subset previously described[16]. While investigating the function of the LP eosinophils, we unexpectedly found that the LP eosinophils could produce ATRA and express TGF-β1 mRNA. In particular, both the ALDH activity and ATRA levels produced by the LP eosinophils were significantly higher than for the CD103^+^ DC isolated from murine LP, strongly implying that the LP eosinophils could have the potential to induce differentiation of naïve T cells into Treg cells.

Studies have demonstrated that a specific reduction in Treg cells was observed in the LP of eosinophil-deficient mice[29]. We confirmed these findings in vitro co-culture experiments that LP eosinophils could induce the differentiation of Foxp3^+^ Treg cells.

Compared to the LP eosinophils (CD11c^+^ CD80^+^), the eosinophils from mouse peripheral blood expressed a different phenotype (CD11c^−^CD80^−^) but also lacked the ability to produce ATRA and express TGF-β1 mRNA to induce Treg response.

Eosinophils are produced in the bone marrow from pluripotent stem cells, and then into a separate eosinophil lineage like LP and PB eosinophils. We speculate that the need for the immune system to tolerate the gut microbiota and food in the intestines could account for this functional difference between LP and PB eosinophils. Taken together, this study reveals that LP eosinophils, unlike PB eosinophils, can induce Treg differentiation through a mechanism dependent on ATRA and TGF-β1.
